# Oxygen saturation before and after mechanical thrombectomy and functional outcome in patients with acute ischemic stroke

**DOI:** 10.3389/fcvm.2022.935189

**Published:** 2022-09-30

**Authors:** Shuhong Yu, Shuai Yu, Hang Zhang, Qingyong Dai, Hao Huang, Yi Luo, Zhiliang Guo, Guodong Xiao

**Affiliations:** ^1^Department of Encephalopathy, Suzhou Integrated Traditional Chinese and Western Medicine Hospital, Suzhou, China; ^2^Department of Neurology and Suzhou Clinical Research Center of Neurological Disease, The Second Affiliated Hospital of Soochow University, Suzhou, China; ^3^Department of Neurology, Xishan People’s Hospital of Wuxi City, Wuxi, China

**Keywords:** oxygen saturation, outcome, ischemic reperfusion injury, ischemic penumbra, mechanical thrombectomy

## Abstract

**Background and purpose:**

Currently, there is a lack of effective neuroprotective strategies to break the ceiling effect of mechanical thrombectomy (MT), and one of the most promising is normobaric oxygen treatment. However, the impact of pre- and post-MT oxygen saturation on clinical outcomes in patients with acute ischemic stroke (AIS) remains unclear. We aimed to determine the influence of preoperative and postoperative oxygen saturation on 3-month poor outcome in patients with AIS.

**Methods:**

A total of 239 consecutive stroke patients with successful recanalization by MT between May 2017 and March 2021 were analyzed. Oxygen saturation was measured non-invasively by pulse oximetry at baseline and continually after MT. Regression analysis was used to assess the association of preoperative and postoperative oxygen saturation with a 3-month poor outcome (modified Rankin Scale score: 3–6).

**Results:**

Decreased preoperative oxygen saturation level was associated with an increased risk of poor outcome (odds ratio, 0.85; 95% CI, 0.73–0.98; *P* = 0.0293). Postoperative oxygen saturation had the opposite effect on poor outcome (odds ratio, 1.60; 95% CI, 1.13–2.27; *P* = 0.0088).

**Conclusion:**

Preoperative and postoperative oxygen saturation have different impacts on 3-month poor outcome in patients with AIS with successful recanalization by MT.

## Introduction

Mechanical thrombectomy (MT) is currently the standard treatment for acute ischemic stroke (AIS) caused by large vessel occlusion (1). Despite high rates of recanalization and optimal patient selection, approximately half of the treated patients may not achieve functional independence at 3 months. This is largely attributable to ischemic core expansion due to the lack of timely neuroprotection before recanalization and ischemic reperfusion injury after recanalization (1–3). One of the main mechanisms of ischemic reperfusion injury is damage caused by oxygen-free radicals (3, 4). Theoretically, there is sufficient blood flow and oxygen after reperfusion, and excessive oxygen may potentiate brain injury by triggering the production of free radicals and facilitating vasoconstriction-induced hypoperfusion (4, 5). Recent studies have also demonstrated that higher oxygen saturation after recanalization is associated with higher odds of mortality (6, 7).

In terms of the influence of preoperative oxygen saturation on the 3-month poor outcome, previous studies reported that the essence of ischemic core expansion before recanalization is severe persistent hypoxia (2, 3, 8). Improving oxygen supply to the penumbra would be expected to maintain the penumbra until the occurrence of reperfusion, thus reducing the final damage (3, 8). In many studies, preoperative normobaric oxygen (NBO) treatment was found to improve tissue oxygenation and reduce blood-brain barrier damage, cerebral infarction volume, and neurological impairment scores (9, 10). These findings suggest that higher oxygen saturation before recanalization may reduce the risk of poor outcome. However, the impact of pre- and post-MT oxygen saturation on the functional outcomes in patients with AIS is not well characterized in the contemporary literature.

We aimed to assess the relationship of pre- and post-MT oxygen saturation levels with clinical outcomes after MT in patients with AIS. Our findings may help unravel the precise roles of oxygen treatment before and after recanalization and provide novel insights for further improvements in neuroprotective interventions, such as NBO treatment (3, 8).

## Materials and methods

### Study design and participants

Consecutive patients with AIS receiving endovascular treatment at the department of neurology at our hospital between December 2017 and March 2021 were prospectively recruited. The inclusion criteria for the retrospective analysis were as follows: (1) age ≥ 18 years; (2) clinical diagnosis of AIS with anterior or posterior circulation large vessel occlusion; (3) underwent first-line treatment with direct aspiration, stent retriever, or a combination of a stent retriever and local aspiration catheter; and (4) achievement of successful recanalization. The exclusion criteria were as follows: (1) patients treated with intra-arterial thrombolysis only (*n* = 56); (2) chronic obstructive pulmonary disease, lung inflammation, pleural effusion, pneumothorax, acute respiratory distress syndrome, irregular respiratory movements, decompensated congestive cardiac failure, reduced peripheral perfusion leading to unobtainable or poor oximetry trace, severe hepatic or renal dysfunction (*n* = 8); (3) patients without successful recanalization (*n* = 39); and (4) incomplete oxygen saturation, clinical data, or patients lost to follow-up (*n* = 55). A total of 239 patients with AIS qualified the study-selection criteria and were included in the analysis (flowchart of participant selection: Supplementary Figure I in Supplementary Appendix A). The study protocol was approved by the Ethics Committee of the Second Affiliated Hospital of Soochow University, and written informed consent was obtained from all patients or their relatives.

### Clinical protocol and laboratory tests

Medical history including demographics (age and sex), potential risk factors for stroke (atrial fibrillation, hypertension, diabetes, hyperlipidemia, smoking, and alcohol use), stroke etiology, stroke severity, admission Alberta Stroke Program Early CT Score (ASPECTS), pretreatment with intravenous thrombolysis (IVT), premorbid modified Rankin Scale (mRS) score, occlusion site, the status of the collateral circulation, symptom onset (or when last seen well) to reperfusion time, blood indices, 12-lead electrocardiogram, and chest radiography/CT were performed at admission. The risk factors for stroke were defined according to previous published criteria (11). The etiological subtypes and stroke severity were recorded as previously described (12). The status of the collateral circulation before thrombectomy was evaluated using the American Society of Interventional and Therapeutic Neuroradiology/Society of Interventional Radiology scale (13). Reperfusion status was graded in the final angiogram according to the modified Thrombolysis in Cerebral Infarction (mTICI) score, with successful recanalization defined as a score of 2b or 3 (12). Patients were followed up at 3 months telephonically or during an outpatient visit. Poor outcome was defined as a modified Rankin Scale score of 3–6 at month 3 after admission.

### Assessment of oxygen saturation

Oxygen saturation was measured non-invasively by pulse oximetry using a patient monitor (Philips Medizin Systeme Boeblingen GmbH, Boeblingen, Germany) (14, 15). Only a single datum of preoperative oxygen saturation was recorded in some patients due to the different times of stay in the emergency treatment room. Therefore, we used the first record after admission as preoperative oxygen saturation. In addition, most patients had multiple oxygen saturation records during the first 6 h following MT. Therefore, we used the median levels of oxygen saturation after recanalization (7 records were used for the current analysis: first record, 1 h, 2 h, 3 h, 4 h, 5 h, and 6 h after recanalization) as postoperative oxygen saturation.

### Statistical analysis

Characteristics of patients with and without poor outcome were compared using the chi-squared test, Fisher exact test, or Mann-Whitney U test. The multivariate analysis regression model was used to evaluate the association of pre- and post-MT oxygen saturation with a 3-month poor outcome. We first included age and female sex in the model (model 1). Subsequently, variables were included in model 2 if they were associated with poor outcome (*P* < 0.10) or changed the estimated effect of oxygen saturation on poor outcome by more than 10%. The association of each confounder with the outcomes of interest is shown in Supplementary Tables II–VI in Supplementary Appendix A (16, 17).

The generalized additive models (GAM) and two-piecewise linear regression model were used to identify the non-linear relationships and calculate the threshold effect of oxygen saturation on poor outcome. The likelihood ratio tests were used to evaluate the modifications and interactions between oxygen saturation and subgroup variables on the poor outcome. Receiver operating characteristic (ROC) curve analysis was performed to assess the overall discriminative ability of oxygen saturation for poor outcome and to establish an optimal cutoff point based on the Youden index. All statistical analyses were performed using EmpowerStats^1^ (X&Y Solutions, Inc., Boston, MA). Two-sided *P* values < 0.05 were considered indicative of statistical significance (16, 17).

## Results

### Baseline characteristics of patients

Except for the higher premorbid mRS in excluded patients, most of the baseline characteristics were balanced between the patients included and excluded from this study (Supplementary Table I in Supplementary Appendix A). A total of 239 patients with AIS (median age: 68 years) with successful recanalization by MT were included in this study. The main baseline characteristics of study participants according to the development of poor outcome are presented in Table 1. Participants with poor outcome tended to be older, female, and had higher baseline NIHSS scores, number of passes, history of hypertension, smoking, and lower ASPECTS and collateral scores. These patients were also more likely to have lower preoperative oxygen saturation but higher median levels of postoperative oxygen saturation (Table 1).

**TABLE 1:** Baseline characteristics of study participants according to the development of poor outcome.

Characteristics	All patients	mRS 0-2	mRS 3-6	*P*-value
No. of patients	238	96	143	
Age, y; median (IQR)	68.00 (58.00−75.00)	64.00 (48.00−72.00)	70.00 (62.00−78.00)	< 0.001
Female, n (%)	64 (51.20%)	32 (33.33%)	72 (50.35%)	0.009
Atrial fibrillation, n (%)	113 (90.40%)	34 (35.42%)	68 (47.55%)	0.063
Hypertension, n (%)	34 (27.20%)	57 (59.38%)	103 (72.03%)	0.041
Diabetes, n (%)	36 (28.80%)	11 (11.46%)	29 (20.28%)	0.073
Hyperlipidemia, n (%)	26 (20.80%)	36 (37.50%)	41 (28.67%)	0.152
History of stroke, n (%)	16 (12.80%)	9 (9.38%)	25 (17.48%)	0.079
Smoking, n (%)	26 (20.80%)	41 (42.71%)	42 (29.37%)	0.034
Drinking, n (%)	14 (11.20%)	30 (31.25%)	29 (20.28%)	0.054
Baseline NIHSS, median (IQR)	16.00 (12.00−19.00)	13.00 (11.00−17.00)	17.00 (14.00−20.00)	< 0.001
ASPECTS, median (IQR)	7.00 (7.00−7.00)	7.00 (7.00−8.00)	7.00 (6.00−7.00)	< 0.001
Occluded artery, n (%)				0.409
ICA	41 (17.15%)	12 (12.50%)	29 (20.28%)	
M1 of the MCA	150 (62.76%)	64 (66.67%)	86 (60.14%)	
Posterior circulation	29 (12.13%)	11 (11.46%)	18 (12.59%)	
Others	19 (7.95%)	9 (9.38%)	10 (6.99%)	
IVT, n (%)	74 (30.96%)	32 (33.33%)	42 (29.37%)	0.516
Premorbid mRS, median (IQR)	0.00 (0.00−0.00)	0.00 (0.00−0.00)	0.00 (0.00−0.00)	0.620
Stroke etiology, n (%)				0.254
LAA	107 (44.77%)	43 (44.79%)	64 (44.76%)	
Cardioembolic	119 (49.79%)	45 (46.88%)	74 (51.75%)	
Others	13 (5.44%)	8 (8.33%)	5 (3.50%)	
Collateral score, median (IQR)	0.00 (0.00−2.00)	1.00 (0.00−2.25)	0.00 (0.00−1.00)	< 0.001
OTR, median (IQR), min	356.00 (281.50−447.50)	356.00 (280.00−435.00)	358.00 (283.50−459.50)	0.872
Number of passes, median (IQR)	2.00 (1.00−3.00)	1.00 (1.00−2.00)	2.00 (1.00−3.00)	0.025
Preoperative oxygen saturation, median (IQR)	99.00 (97.00−100.00)	99.00 (98.00−100.00)	99.00 (96.50−100.00)	0.094
Median levels of postoperative oxygen saturation, median (IQR)	99.00 (98.00−100.00)	98.75 (98.00−99.38)	99.00 (98.00−100.00)	0.010

ASPECTS, Alberta Stroke Program Early CT Score; IQR, interquartile range; ICA, internal carotid artery; IVT, intravenous thrombolysis; LAA, large-artery atherosclerosis; MCA, middle cerebral artery; mRS, modified Rankin Scale; mTICI, modified Thrombolysis in Cerebral Infarction; NIHSS, National Institutes of Health Stroke Scale; OTR, onset to reperfusion time; Posterior circulation, including basilar artery and intracranial part of the vertebral artery.

### Association between oxygen saturation before mechanical thrombectomy and poor outcome

**FIGURE 1:**
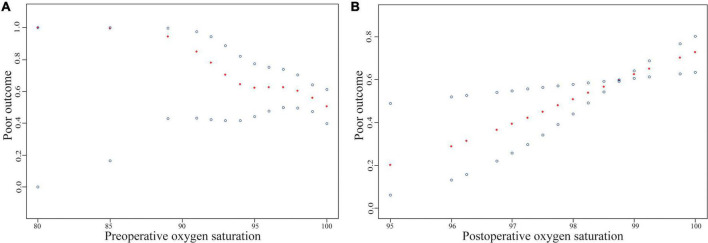
Relationship between preoperative/postoperative oxygen saturation and functional outcomes of ischemic stroke. The red dots in the middle of the figure represent the smooth curve fit, and the blue dots represent the 95% confidence interval of the fit. The scatter plot of curve fit results mainly reflects the probability of 3-month poor outcome (Y-axis) with different oxygen saturation levels (X-axis). Adjusted for variables that were significantly associated with poor outcome (*P* < 0.10) or changed the estimates of oxygen saturation on poor outcome by more than 10% (Supplementary Tables II–VI in Supplementary Appendix A).

Table 2 summarizes the results of the multivariate regression model depicting the relationship between preoperative oxygen saturation and functional outcome. Generalized linear analysis revealed an independent association of preoperative oxygen saturation with a lower mRS score after adjusting for potential confounders (β, −0.04; 95% CI, −0.07 to −0.00; *P* = 0.0421). Multivariate logistic regression analysis also revealed that preoperative oxygen saturation was independently associated with poor outcome (mRS score 3–6) after adjusting for potential confounders (odds ratio [OR], 0.85; 95% CI, 0.73–0.98; *P* = 0.0293; Table 2). Non-linear relationships were not found between preoperative oxygen saturation and functional outcome in the generalized additive models, and the probability of a 3-month poor outcome was reduced with increased preoperative oxygen saturation (Figure 1A). Moreover, there was no statistically significant inflection point in the threshold effect analysis (*P* for the log-likelihood ratio test was 0.159 for poor outcome, which suggests that the standard linear regression rather than a two-piecewise linear regression may be more appropriate to analyze the relationship between preoperative oxygen saturation and poor outcome; Table 3). Subgroup analyses further confirmed these associations between preoperative oxygen saturation and functional outcome (Supplementary Table VII in Supplementary Appendix A).

**TABLE 2:** Relationship between preoperative, postoperative, or combined oxygen saturation and functional outcomes among patients with acute ischemic stroke in different models.

Variable	Non-adjusted model		Model 1		Model 2	
	β/OR (95%CI)	*P*-value	β/OR (95%CI)	*P*-value	β/OR (95%CI)	*P*-value
mRS						
Preoperative oxygen saturation	−0.04 (−0.08, 0.00)	0.0549	−0.05 (−0.09, −0.01)	0.0202	−0.04 (−0.07, −0.00)	0.0421
Postoperative oxygen saturation	0.36 (0.12, 0.60)	0.0030	0.35 (0.13, 0.57)	0.0022	0.23 (0.03, 0.43)	0.0252
Combined preoperative and postoperative oxygen saturation						
HL	0		0		0	
LL	−0.12 (−0.87, 0.63)	0.7633	−0.22 (−0.91, 0.48)	0.5450	0.15 (−0.49, 0.80)	0.6453
HH	0.60 (−0.08, 1.28)	0.0872	0.62 (−0.01, 1.26)	0.0557	0.54 (−0.06, 1.14)	0.0800
LH	1.13 (0.39, 1.87)	0.0031	1.02 (0.34, 1.71)	0.0038	0.84 (0.19, 1.49)	0.0123
*P* for trend	0.0006		0.0004		0.0053	
Poor outcome (mRS score 3-6)						
Preoperative oxygen saturation	0.88 (0.78, 0.99)	0.0373	0.92 (0.81, 1.04)	0.1827	0.85 (0.73, 0.98)	0.0293
Postoperative oxygen saturation	1.41 (1.10, 1.81)	0.0072	1.46 (1.12, 1.91)	0.0058	1.60 (1.13, 2.27)	0.0088
Combined preoperative and postoperative oxygen saturation						
HL	1		1		1	
LL	0.93 (0.43, 2.00)	0.8445	0.84 (0.37, 1.91)	0.6758	1.68 (0.59, 4.80)	0.3328
HH	1.58 (0.78, 3.20)	0.2034	1.75 (0.82, 3.76)	0.1510	2.70 (0.98, 7.39)	0.0538
LH	3.58 (1.55, 8.30)	0.0029	3.87 (1.56, 9.55)	0.0034	6.35 (1.97, 20.52)	0.0020
*P* for trend	0.0013		0.0010		0.0014	

Non-adjusted model: we did not adjust other covariates. Model 1: we adjusted age and female sex. Model 2: we adjusted variables that were significantly associated with outcomes of interest (*P* < 0.10) or changed the estimates of oxygen saturation on outcomes of interest by more than 10% (Supplementary Tables II–VI in Supplementary Appendix A). HL, higher preoperative oxygen saturation and lower postoperative oxygen saturation; LL, lower preoperative oxygen saturation and lower postoperative oxygen saturation; HH, higher preoperative oxygen saturation and higher postoperative oxygen saturation; LH, lower preoperative oxygen saturation and higher postoperative oxygen saturation; mRS, modified Rankin Scale; OR, odds ratio.

**TABLE 3:** The results of two-piecewise linear regression model.

Exposure:	Preoperative oxygen saturation	Postoperative oxygen saturation
Fitting model by standard linear regression	0.85 (0.73, 0.98) 0.0293	1.60 (1.13, 2.27) 0.0088
Fitting model by two-piecewise linear regression		
Inflection point of oxygen saturation (K)	94	99.75
< K	0.50 (0.15, 1.62) 0.2484	1.81 (1.11, 2.97) 0.0183
≥ K	0.93 (0.76, 1.14) 0.4752	0.36 (0.01, 21.86) 0.6244
*P* for log likelihood ratio test	0.159	0.474

Effect: preoperative/postoperative oxygen saturation; Cause: poor outcome; Adjusted: variables that were significantly associated with outcomes of interest (*P* < 0.10) or changed the estimates of oxygen saturation on outcomes of interest by more than 10% (Supplementary Tables II–VI in Supplementary Appendix A).

### Association between oxygen saturation after mechanical thrombectomy and poor outcome

In terms of the relationship between postoperative oxygen saturation and functional outcome, the generalized linear analysis revealed that postoperative oxygen saturation was independently associated with a lower mRS score after adjusting for potential confounders (β, 0.23; 95% CI, 0.03–0.43; *P* = 0.0252). Multivariate logistic regression analysis also revealed that postoperative oxygen saturation was independently associated with poor outcome after adjusting for potential confounders (OR, 1.60; 95% CI, 1.13–2.27; *P* = 0.0088; Table 2). Non-linear relationships were also not found between postoperative oxygen saturation and functional outcome in the generalized additive models, and the probability of poor outcome increased with the increase in postoperative oxygen saturation (Figure 1B). Moreover, there was no statistically significant inflection point in the threshold effect analysis (*P* for the log-likelihood ratio test was 0.474 for poor outcome; Table 3). Subgroup analyses also further confirmed these associations between postoperative oxygen saturation and functional outcome (Supplementary Table VIII in Supplementary Appendix A). These results suggest that the preoperative and postoperative oxygen saturation have different impacts on 3-month poor outcome.

### Combined effect of preoperative and postoperative oxygen saturation on poor outcome

To evaluate the influence of combined preoperative and postoperative oxygen saturation on the poor outcome, patients with AIS were divided into 4 groups according to the median levels of oxygen saturation before and after MT (both were 99%): HL [higher preoperative oxygen saturation (≥ 99%) and lower postoperative oxygen saturation (< 99%)], LL (lower preoperative oxygen saturation and lower postoperative oxygen saturation), HH (higher preoperative oxygen saturation and higher postoperative oxygen saturation), and LH (lower preoperative oxygen saturation and higher postoperative oxygen saturation). LH was associated with a 6.35-fold increase in the risk of poor outcome in comparison to HL after adjusting for all potential confounders (OR, 6.35; 95% CI, 1.97–20.52; *P* trend = 0.0014; Table 2). In addition, ROC curves were used to compare the predictive ability of combined preoperative and postoperative oxygen saturation and preoperative or postoperative oxygen saturation alone on the poor outcome. The combined group showed greater discriminative ability than preoperative alone but not postoperative alone (combined vs. preoperative: 0.583 vs. 0.535, *P* = 0.0033; combined vs. postoperative: 0.583 vs. 0.598, *P* = 0.7134; preoperative vs. postoperative: 0.535 vs. 0.598, *P* = 0.2022). ROC curve analysis was also performed to assess the optimal cutoff level of oxygen saturation to discriminate poor outcome. The optimal preoperative oxygen saturation cutoff level of 97% showed 34.27% sensitivity and 78.12% specificity for distinguishing the presence/absence of poor outcome. The optimal postoperative oxygen saturation cutoff level of 98.75% was associated with 64.34% sensitivity and 55.21% specificity.

## Discussion

In this study, decreased preoperative oxygen saturation level was associated with an increased risk of poor outcome. However, postoperative oxygen saturation had the opposite effect on the poor outcome. Besides, the combined preoperative and postoperative oxygen saturation (LH) was associated with a 6.35-fold increase in the risk of poor outcome in comparison to HL. These results provide novel insights for uncovering the precise roles of oxygen treatment before and after recanalization, providing insights and references for further study of neuroprotective strategies, such as NBO (3, 8).

In previous studies, prophylactic low-dose oxygen supplementation was not found to reduce death or disability in patients with AIS and receiving room air during the first 24 h after stroke was associated with better outcome after 7 months compared to supplemental oxygen therapy with 100% oxygen (18, 19). Furthermore, in recent clinical studies, higher oxygen saturation after recanalization was found to be associated with higher odds of mortality (6, 7). In addition, arterial hyperoxia was independently associated with in-hospital death as compared with either normoxia or hypoxia in ventilated stroke patients admitted to the ICU (7). These studies, however, did not explore the influence of postoperative oxygen saturation on a 3-month poor outcome. To the best of our knowledge, this is the first study to explore the association between postoperative oxygen saturation and poor outcome. We found that higher postoperative oxygen saturation was associated with poor outcome, which suggests that additional oxygen supplements may not be required after recanalization.

To the best of our knowledge, no studies have investigated the influence of preoperative oxygen saturation on the poor outcome. In this study, decreased preoperative oxygen saturation level was associated with an increased risk of poor outcome, which was contrary to the effect of postoperative oxygen saturation. These results may provide novel ideas for unraveling the optimal duration of oxygen treatment. Moreover, administering oxygen inhalation before surgery but no additional oxygen supplement after recanalization may be a better treatment strategy. In addition, the combined group showed greater discriminative ability than preoperative alone but not postoperative; this may possibly be attributable to the small sample size in our study, which may have weakened the statistical power. Therefore, further studies with larger samples are required to draw more definitive conclusions.

The mechanisms by which the effect of preoperative oxygen saturation on poor outcome differs from that of postoperative oxygen saturation have not yet been clarified, but they seem to be related to the complex roles of oxygen in ischemic injury. Prior to the restoration of blood flow, the essence of ischemic core expansion is severe persistent hypoxia (2, 3, 8). Lower oxygen levels at this moment may be related to more core growth. After the restoration of blood flow, ischemic reperfusion injury, rather than hypoxia, is the primary consideration. Hyperoxia during the reperfusion period may induce the production of ROS, which aggravates the reperfusion injury. In addition, post-recanalization hyperoxia induces vasoconstriction due to the inactivation of nitric oxide by ROS superoxide anion (4, 5). Therefore, higher preoperative oxygen saturation and lower postoperative oxygen saturation (HL) may be the best practices for stroke patients who have undergone successful recanalization by MT. We also found that LH was associated with a 6.35-fold increase in the risk of poor outcome in comparison to HL. Recently, a study attempted to determine the treatment window for oxygen therapy, i.e., the optimal timing of the start of NBO treatment and the optimal duration of treatment (10). In the 60-min MCAO rats, the T2-derived lesion volume in the air, 25-min NBO, and 150-min NBO groups increased by 19%, 4%, and -18%, respectively. However, CBF was differentially higher in the 25-min NBO group compared to air or 150-min NBO groups at 180 min in the mismatch CBF data, which might be related to vasoconstriction-induced hypoperfusion of the 150-min NBO treatment (5, 10). Similar results were observed in the ADC data. The 25-min NBO group showed a trend of reducing ADC lesion volume compared to air or 150-min NBO groups. In addition, the study also had some limitations. First, the study lacked a postoperative NBO group (treated with NBO after reperfusion), which would have helped separate the neuroprotective role of 150-min NBO treatment by comparison with the air group. Because the beneficial role of 150-min NBO was possibly contributed by NBO treatment before reperfusion, post-reperfusion NBO treatment may have had no additional neuroprotective effect. Further studies are required to test this hypothesis. Second, the NBO treatment time was relatively insufficient before reperfusion, which may have caused the ‘freezing’ penumbra having not been fully played. Third, this study did not perform behavioral assessments and histological validation (10). Thus, further studies are required to explore the different roles of preoperative and postoperative oxygen treatment and their mechanisms.

The main strength of our study is that we comprehensively evaluated the influence of preoperative and postoperative oxygen saturation on 3-month poor outcome in stroke patients with successful recanalization by MT. Nonetheless, this study has some limitations. First, this was a retrospective single-center study with small sample size. Moreover, our cohort represents a subgroup of stroke patients who achieved successful recanalization by MT treatment; thus, our results may not be generalizable to the whole population of stroke patients, and further studies from other samples of patients with AIS are required to validate our results. Second, the oxygen saturation was measured non-invasively by pulse oximetry at irregular times. However, a recent study showed a strong correlation between daytime and nighttime results, and borderline hypoxia during the day was found to be strongly predictive of overt hypoxia at night (20). Moreover, we used multiple postoperative oxygen saturation recordings for the current analysis according to the actual situation. Nonetheless, further studies should include arterial blood gas analysis at multiple time points. Third, the influence of HL was likely confounded by complications (e.g., aspiration pneumonia) as a cause for developing hypoxia in the hospital and poor outcome, and this does not appear to be adjusted for in all the models. Theoretically, patients with pneumonia complications may develop hypoxia, and these patients are also more likely to develop a poor outcome. However, our results were not consistent with the theorized expectations. We found that HL (with lower postoperative oxygen saturation) was associated with better clinical outcomes. Therefore, the pneumonia complications may not have had a major confounding influence on the independent correlation between HL and poor outcome. In addition, we found that the significant association of HL with poor outcome was independent of pneumonia complications in the multivariate analysis (Supplementary Table IX in Supplementary Appendix A) when pneumonia complications were included as confounding factors, which did not change the main results of our study. Nevertheless, it is noteworthy that this is the first study showing the complex relationships between oxygen saturation and poor outcome in patients with AIS experiencing MT. In this regard, attempts to maintain higher preoperative but not postoperative oxygen saturation have important implications for stroke outcome.

## Conclusion

We found that the preoperative and postoperative oxygen saturation have different impacts on 3-month poor outcome in patients with AIS with successful recanalization by MT. Decreased preoperative oxygen saturation level was associated with an increased risk of poor outcome. However, decreased postoperative oxygen saturation level was associated with decreased risk of poor outcome.

## Data availability statement

The original contributions presented in this study are included in the article/Supplementary material, further inquiries can be directed to the corresponding author/s.

## Ethics statement

The studies involving human participants were reviewed and approved by the Ethics Committee of the Second Affiliated Hospital of Soochow University. The patients/participants provided their written informed consent to participate in this study.

## Author contributions

SHY, SY, HZ, GX, and ZG contributed to the concept and rationale for the study. SHY, SY, and HZ were responsible for the first draft. ZG contributed to the statistical analyses. YL and HH performed the data collection. ZG, GX, and QD contributed to the first revision. All authors read and approved the final manuscript.

## Footnotes

1
http://www.empowerstats.com
