# Supplementary material for: Oxygen saturation before and after mechanical thrombectomy and functional outcome in patients with acute ischemic stroke
Source: Front Cardiovasc Med. 2022 Sep 30;9:935189. doi: 10.3389/fcvm.2022.935189 (PMC9575944; doi:10.3389/fcvm.2022.935189)
Supplement: Supplementary file 1 [file Data_Sheet_1.doc]

**Appendix A**

Oxygen saturation before and after mechanical thrombectomy and functional outcome in patients with acute ischemic stroke

Table I. Demographic and clinical characteristics of included and excluded patients

| Characteristics | Patients included | Patients excluded | *P* Value |
| --- | --- | --- | --- |
| No. of patients | 239 | 158 |  |
| Age, y; median (IQR) | 68.00 (58.00-75.00) | 68.00 (55.25-76.00) | 0.979 |
| Female, n (%) | 104 (43.51%) | 61 (38.61%) | 0.332 |
| Atrial fibrillation, n (%) | 102 (42.68%) | 64 (40.51%) | 0.668 |
| Hypertension, n (%) | 160 (66.95%) | 120 (75.95%) | 0.054 |
| Diabetes, n (%) | 40 (16.74%) | 33 (20.89%) | 0.296 |
| Hyperlipidemia, n (%) | 77 (32.22%) | 60 (37.97%) | 0.238 |
| History of stroke, n (%) | 34 (14.23%) | 29 (18.35%) | 0.270 |
| Smoking, n (%) | 83 (34.73%) | 46 (29.11%) | 0.242 |
| Drinking, n (%) | 59 (24.69%) | 34 (21.529%) | 0.683 |
| Baseline NIHSS, median (IQR) | 16.00 (12.00-19.00) | 16.00 (12.00-20.00) | 0.702 |
| IVT, n (%) | 74 (30.96%) | 52 (32.91%) | 0.683 |
| Stroke etiology, n (%) |  |  | 0.487 |
| LAA | 107 (44.77%) | 79 (50.00%) |  |
| Cardioembolic | 119 (49.79%) | 69 (43.67%) |  |
| Others | 13 (5.44%) | 10 (6.33%) |  |
| Premorbid mRS, median (IQR) | 0.00 (0.00-0.00) | 0.00 (0.00-0.00) | 0.019 |

IQR, interquartile range; IVT, intravenous thrombolysis; LAA, large-artery atherosclerosis; mRS, modified Rankin Scale; NIHSS, National Institutes of Health Stroke Scale.

**Table II.** The collinearity screening of baseline characteristics

| Characteristics | Variance inflation factor |
| --- | --- |
| Age | 1.7 |
| Female | 2 |
| Atrial fibrillation | 2.1 |
| Hypertension | 1.3 |
| Diabetes | 1.1 |
| Hyperlipidemia | 1.2 |
| History of stroke | 1.6 |
| Smoking | 2.2 |
| Drinking | 1.8 |
| Baseline NIHSS | 1.2 |
| ASPECTS | 1.2 |
| Occluded artery | 1.2 |
| IVT | 1.1 |
| Premorbid mRS | 1.6 |
| Stroke etiology | 1.8 |
| Collateral score | 1.3 |
| OTR | 1.2 |
| Number of passes | 1.2 |
| Preoperative oxygen saturation | 1.2 |
| Postoperative oxygen saturation | 2.2 |
| Combined preoperative and postoperative oxygen saturation | 2.4 |

ASPECTS; Alberta Stroke Program Early CT Score; IVT, intravenous thrombolysis; mRS, modified Rankin Scale; NIHSS, National Institutes of Health Stroke Scale; OTR, onset to reperfusion time.

We think collinearity exists and eliminate these covariables in the final models if their variance inflation factors are greater than or equal to 5.

**Table III. Associations of covariates with poor outcome**

| Covariates | exp(beta) | 95%CI | *P* Value |
| --- | --- | --- | --- |
| Age | 1.0545 | 1.0321-1.0773 | <0.0001 |
| Female | 2.0282 | 1.1864-3.4672 | 0.0097 |
| Atrial fibrillation | 1.6533 | 0.9715-2.8136 | 0.0638 |
| Hypertension | 1.7618 | 1.0195-3.0446 | 0.0424 |
| Diabetes | 1.9657 | 0.9297-4.1561 | 0.0769 |
| Hyperlipidemia | 0.6699 | 0.3867-1.1607 | 0.1532 |
| History of stroke | 2.0480 | 0.9105-4.6069 | 0.0831 |
| Smoking | 0.5578 | 0.3246-0.9585 | 0.0346 |
| Drinking | 0.5596 | 0.3091-1.0133 | 0.0553 |
| Baseline NIHSS | 1.1412 | 1.0753-1.2111 | <0.0001 |
| ASPECTS | 0.5395 | 0.3768-0.7723 | 0.0007 |
| Occluded artery |  |  |  |
| ICA | Reference | Reference | Reference |
| M1 of the MCA | 0.5560 | 0.2636-1.1730 | 0.1233 |
| Posterior circulation | 0.6771 | 0.2472-1.8546 | 0.4482 |
| Others | 0.4598 | 0.1494-1.4149 | 0.1755 |
| IVT | 0.8317 | 0.4768-1.4508 | 0.5162 |
| Premorbid mRS | 1.2746 | 0.6522-2.4909 | 0.4779 |
| Stroke etiology |  |  |  |
| LAA | Reference | Reference | Reference |
| Cardioembolic | 1.1049 | 0.6468-1.8872 | 0.7151 |
| Others | 0.4199 | 0.1287-1.3698 | 0.1503 |
| Collateral score | 0.4688 | 1.3698-0.6125 | <0.0001 |
| OTR | 0.9994 | 0.9980-1.0007 | 0.3650 |
| Number of passes | 1.2975 | 0.9998-1.6839 | 0.0502 |

ASPECTS, Alberta Stroke Program Early CT Score; ICA, internal carotid artery; IVT, intravenous thrombolysis; LAA, large-artery atherosclerosis; MCA, middle cerebral artery; mRS, modified Rankin Scale; NIHSS, National Institutes of Health Stroke Scale; OTR, onset to reperfusion time; Posterior circulation, including basilar artery and intracranial part of the vertebral artery.

**Table IV. The adjusting roles of potential confounders on the estimates of preoperative oxygen saturation on poor outcome**

| +/- covariates | Basic model | Complete model | The selected covariates |
| --- | --- | --- | --- |
| Initial regression coefficient of preoperative oxygen saturation | -0.1229 | -0.1669 |  |
| Age | -0.0782 * | -0.1884 * | Yes |
| Female, | -0.1166 | -0.1659 |  |
| Atrial fibrillation | -0.1203 | -0.1614 |  |
| Hypertension | -0.1157 | -0.1668 |  |
| Diabetes | -0.1195 | -0.1640 |  |
| Hyperlipidemia | -0.1273 | -0.1648 |  |
| History of stroke | -0.1177 | -0.1699 |  |
| Smoking | -0.1132 | -0.1712 |  |
| Drinking | -0.1170 | -0.1671 |  |
| Baseline NIHSS | -0.1699 * | -0.1229 * | Yes |
| ASPECTS | -0.1330 | -0.1656 |  |
| Occluded artery | -0.1160 | -0.1691 |  |
| IVT | -0.1248 | -0.1673 |  |
| Premorbid mRS | -0.1228 | -0.1673 |  |
| Stroke etiology | -0.1172 | -0.1617 |  |
| Collateral score | -0.1604 * | -0.1441 * | Yes |
| OTR | -0.1219 | -0.1670 |  |
| Number of passes | -0.1256 | -0.1681 |  |

* These confounders changed the estimates of oxygen saturation on poor outcome by more than 10% when introduce covariates into the basic model or remove covariates from the complete model.

**Table V.** The selected covariates

| Y | X | The selected covariates（Criterion 1） | The selected covariates（Criterion 2） |
| --- | --- | --- | --- |
| mRS score | Preoperative oxygen saturation | Age, Diabetes, Smoking, Baseline NIHSS, Occluded artery, Collateral score | Age, Female, Atrial fibrillation, Hypertension, Diabetes, Smoking, Drinking, Baseline NIHSS, ASPECTS, Occluded artery, TOAST, Collateral score, OTR, Number of passes |
| Poor outcome | Preoperative oxygen saturation | Age, Baseline NIHSS, Collateral score | Age, Female, Atrial fibrillation, Hypertension, Diabetes, History of stroke, Smoking, Drinking, Baseline NIHSS, ASPECTS, Collateral score, Number of passes |

Criterion 1: These confounders changed the estimates of oxygen saturation on the outcomes of interest by more than 10% when introduce covariates into the basic model or remove covariates from the complete model.

Criterion 2: These variables were significantly associated with mRS score/poor outcome (*P* < 0.10) or changed the estimates of oxygen saturation on the outcomes of interest by more than 10%.

**Table VI.** The selected covariates

| Y | X | The selected covariates（Criterion 1） | The selected covariates（Criterion 2） |
| --- | --- | --- | --- |
| mRS score | Postoperative oxygen saturation | Age, Baseline NIHSS, Occluded artery, Collateral score | Age, Female, Atrial fibrillation, Hypertension, Diabetes, Smoking, Drinking, Baseline NIHSS, ASPECTS, Occluded artery, TOAST, Collateral score, Number of passes |
| Poor outcome | Postoperative oxygen saturation | Age, Baseline NIHSS | Age, Female, Atrial fibrillation, Hypertension, Diabetes, History of stroke, Smoking, Drinking, Baseline NIHSS, ASPECTS, Collateral score, Number of passes |
| mRS score | Combined preoperative and postoperative oxygen saturation | Age, Baseline NIHSS | Age, Female, Atrial fibrillation, Hypertension, Diabetes, Smoking, Drinking, Baseline NIHSS, ASPECTS, Occluded artery, TOAST, Collateral score, Number of passes |
| Poor outcome | Combined preoperative and postoperative oxygen saturation | Age | Age, Female, Atrial fibrillation, Hypertension, Diabetes, History of stroke, Smoking, Drinking, Baseline NIHSS, ASPECTS, Collateral score, Number of passes |

Criterion 1: These confounders changed the estimates of oxygen saturation on the outcomes of interest by more than 10% when introduce covariates into the basic model or remove covariates from the complete model.

Criterion 2: These variables were significantly associated with mRS score/poor outcome (*P* < 0.10) or changed the estimates of oxygen saturation on the outcomes of interest by more than 10%.

**Table VII. Subgroup analyses on the association between preoperative oxygen saturation and functional outcome**

| Subgroup | N | mRS | | | Poor outcome (mRS score 3-6) | | |
| --- | --- | --- | --- | --- | --- | --- | --- |
|  |  | β(95%CI) | *P* value | *P* for interaction | OR (95%CI) | *P* value | *P* for interaction |
| Age, years |  |  |  | 0.0120 |  |  | 0.5667 |
| ＜65 | 98 | -0.00 (-0.04, -0.04) | 0.8871 |  | 0.85 (0.61, 1.18) | 0.1319 |  |
| ≥65 | 141 | -0.13 (-0.23, -0.04) | 0.0067 |  | 0.80 (0.66, 0.97) | 0.0248 |  |
| Sex |  |  |  | 0.0752 |  |  | 0.6590 |
| Male | 135 | -0.01 (-0.05, 0.03) | 0.5798 |  | 0.79 (-0.62, 1.01) | 0.0591 |  |
| Female | 104 | -0.12 (-0.23, -0.01) | 0.0353 |  | 0.84 (0.66, 1.07) | 0.1683 |  |
| Atrial fibrillation |  |  |  | 0.1609 |  |  | 0.4729 |
| No | 137 | -0.02 (-0.06, 0.02) | 0.2378 |  | 0.86 (0.68, 1.09) | 0.2152 |  |
| Yes | 102 | -0.15 (-0.30, -0.00) | 0.0531 |  | 0.73 (0.56, 0.95) | 0.0184 |  |
| Hypertension |  |  |  | 0.3093 |  |  | 0.5586 |
| No | 79 | -0.03 (-0.07, 0.01) | 0.2006 |  | 0.71 (0.43, 1.17) | 0.1772 |  |
| Yes | 160 | -0.07 (-0.18, 0.03) | 0.1559 |  | 0.89 (0.75, 1.07) | 0.2071 |  |
| Diabetes |  |  |  | 0.0251 |  |  | 0.9326 |
| No | 199 | -0.12 (-0.21, -0.04) | 0.0057 |  | 0.84 (0.71, 0.99) | 0.0410 |  |
| Yes | 40 | -0.05 (-0.12, 0.02) | 0.1991 |  | 17.85 (0.00, Inf) | 0.9993 |  |
| Hyperlipidemia |  |  |  | 0.0876 |  |  | 0.6871 |
| No | 162 | -0.10 (-0.19, -0.01) | 0.0272 |  | 0.87 (0.72, 1.05) | 0.1786 |  |
| Yes | 77 | -0.00 (-0.05, 0.04) | 0.8722 |  | 0.79 (0.50, 1.25) | 0.3119 |  |
| History of stroke |  |  |  | 0.9407 |  |  | 0.3784 |
| No | 205 | -0.04 (-0.08, -0.00) | 0.0297 |  | 0.83 (0.70, 0.97) | 0.0231 |  |
| Yes | 34 | 0.02 (-0.24, 0.27) | 0.9070 |  | 147.17 (0.00, Inf) | 0.99999 |  |
| Smoking |  |  |  | 0.0964 |  |  | 0.2202 |
| No | 156 | -0.05 (-0.09, -0.01) | 0.0082 |  | 0.00 (0.00, 7.82) | 0.1175 |  |
| Yes | 83 | 0.06 (-0.17, 0.29) | 0.6037 |  | 0.00 (0.00, 0.00) | 0.0100 |  |
| Drinking |  |  |  | 0.7478 |  |  | 0.5396 |
| No | 180 | -0.04 (-0.08, -0.00) | 0.0278 |  | 0.00 (0.00, 0.09) | 0.0127 |  |
| Yes | 59 | -0.24 (-0.55, 0.07) | 0.1379 |  | 0.00 (0.00, Inf) | 0.9997 |  |
| Baseline NIHSS |  |  |  | 0.1396 |  |  | 0.3299 |
| ＜16 | 114 | -0.18 (-0.33, -0.03) | 0.0179 |  | 0.00 (0.00, 0.22) | 0.0270 |  |
| ≥16 | 125 | -0.03 (-0.07, 0.01) | 0.1488 |  | 0.00 (0.00, 0.89) | 0.0480 |  |
| IVT |  |  |  | 0.0791 |  |  | 0.8855 |
| No | 165 | -0.03 (-0.07, 0.01) | 0.1826 |  | 0.85 (0.69, 1.03) | 0.1034 |  |
| Yes | 74 | -0.07 (-0.22, 0.07) | 0.3050 |  | 0.90 (0.61, 1.34) | 0.6139 |  |

In the multivariate models, confounding factors were included unless the variable was used as a stratification variable.

IVT, intravenous thrombolysis; N, No. of participants; NIHSS, National Institutes of Health Stroke Scale; OR, odds ratio.

**Table VIII. Subgroup analyses on the association between postoperative oxygen saturation and functional outcome**

| Subgroup | N | mRS | | | Poor outcome (mRS score 3-6) | | |
| --- | --- | --- | --- | --- | --- | --- | --- |
|  |  | β(95%CI) | *P* value | *P* for interaction | OR (95%CI) | *P* value | *P* for interaction |
| Age, years |  |  |  | 0.5723 |  |  | 0.2299 |
| ＜65 | 98 | 0.33 (-0.01, 0.68) | 0.0607 |  | 2.45 (1.07, 5.60) | 0.0339 |  |
| ≥65 | 141 | 0.18 (-0.10, 0.46) | 0.2101 |  | 1.47 (0.91, 2.37) | 0.1140 |  |
| Sex |  |  |  | 0.3970 |  |  | 0.7283 |
| Male | 135 | 0.14 (-0.15, 0.43) | 0.3462 |  | 1.54 (0.91, 2.62) | 0.1072 |  |
| Female | 104 | 0.25 (-0.07, 0.56) | 0.1364 |  | 1.92 (1.02, 3.62) | 0.0435 |  |
| Atrial fibrillation |  |  |  | 0.5051 |  |  | 0.0880 |
| No | 137 | 0.19 (-0.11, 0.48) | 0.2220 |  | 1.17 (0.67, 2.07) | 0.5798 |  |
| Yes | 102 | 0.27 (-0.04, 0.58) | 0.0898 |  | 2.38 (1.29, 4.40) | 0.00557 |  |
| Hypertension |  |  |  | 0.9821 |  |  | 0.9235 |
| No | 79 | 0.19 (-0.22, 0.59) | 0.3694 |  | 1.44 (0.50, 4.13) | 0.5022 |  |
| Yes | 160 | 0.25 (-0.01, 0.50) | 0.0587 |  | 1.69 (1.11, 2.57) | 0.0141 |  |
| Diabetes |  |  |  | 0.0392 |  |  | 0.0238 |
| No | 199 | 0.15 (-0.07, 0.36) | 0.1762 |  | 1.35 (0.93, 1.96) | 0.1114 |  |
| Yes | 40 | 1.44 (0.43, 2.45) | 0.0118 |  | Inf. (0.00, Inf) | 0.9994 |  |
| Hyperlipidemia |  |  |  | 0.9528 |  |  | 0.6021 |
| No | 162 | 0.24 (-0.01, 0.49) | 0.0641 |  | 1.77 (1.08, 2.89) | 0.0240 |  |
| Yes | 77 | 0.20 (-0.15, 0.55) | 0.2626 |  | 1.55 (0.76, 3.18) | 0.2289 |  |
| History of stroke |  |  |  | 0.9874 |  |  | 0.9196 |
| No | 205 | 0.24 (0.02, 0.04) | 0.0378 |  | 1.62 (1.12, 2.36) | 0.0106 |  |
| Yes | 34 | 0.16 (-0.54, 0.86) | 0.6670 |  | 0.01 (0.00, Inf) | 1.0000 |  |
| Smoking |  |  |  | 0.6366 |  |  | 0.3140 |
| No | 156 | 0.20 (-0.05, 0.45) | 0.1271 |  | 1.66 (1.02, 2.71) | 0.0428 |  |
| Yes | 83 | 0.22 (-0.17, 0.61) | 0.2666 |  | 1.72 (0.90, 3.29) | 0.1005 |  |
| Drinking |  |  |  | 0.7656 |  |  | 0.9646 |
| No | 180 | 0.26 (0.02, 0.49) | 0.0355 |  | 1.70 (1.09, 2.65) | 0.0183 |  |
| Yes | 59 | 0.06 (-0.51, 0.62) | 0.8476 |  | 1.48 (0.67, 3.29) | 0.3331 |  |
| Baseline NIHSS |  |  |  | 0.3609 |  |  | 0.6991 |
| ＜16 | 114 | 0.34 (0.01, 0.68) | 0.0471 |  | 2.19 (1.06, 4.53) | 0.0339 |  |
| ≥16 | 125 | 0.12 (-0.19, 0.44) | 0.4473 |  | 1.40 (0.79, 2.46) | 0.2486 |  |
| IVT |  |  |  | 0.4658 |  |  | 0.6277 |
| No | 165 | 0.32 (0.08, 0.56) | 0.0105 |  | 1.98 (1.24, 3.15) | 0.0041 |  |
| Yes | 74 | 0.09 (-0.32, 0.51) | 0.6590 |  | 1.50 (0.60, 3.78) | 0.3881 |  |

In the multivariate models, confounding factors were included unless the variable was used as a stratification variable.

IVT, intravenous thrombolysis; N, No. of participants; NIHSS, National Institutes of Health Stroke Scale; OR, odds ratio.

**Table IX. Association of oxygen saturation with poor outcome was independent of pneumonia complications**

| Variable | Non-adjusted model | |  | Model 1 | |  | Model 2 | |
| --- | --- | --- | --- | --- | --- | --- | --- | --- |
|  | β/OR (95%CI) | *P* Value |  | β/OR (95%CI) | *P* Value |  | β/OR (95%CI) | *P* Value |
| mRS |  |  |  |  |  |  |  |  |
| HL | 0 |  |  | 0 |  |  | 0 |  |
| LL | -0.12 (-0.87, 0.63) | 0.7633 |  | -0.22 (-0.91, 0.48) | 0.5450 |  | 0.09 (-0.56, 0.73) | 0.7923 |
| HH | 0.60 (-0.08, 1.28) | 0.0872 |  | 0.62 (-0.01, 1.26) | 0.0557 |  | 0.39 (-0.22, 1.00) | 0.2145 |
| LH | 1.13 (0.39, 1.87) | 0.0031 |  | 1.02 (0.34, 1.71) | 0.0038 |  | 0.72 (0.06, 1.37) | 0.0338 |
| *P* for trend | 0.0006 |  |  | 0.0004 |  |  | 0.0053 | 0.0201 |
| Poor outcome (mRS score 3-6) |  |  |  |  |  |  |  |  |
| HL | 1 |  |  | 1 |  |  | 1 |  |
| LL | 0.93 (0.43, 2.00) | 0.8445 |  | 0.84 (0.37, 1.91) | 0.6758 |  | 1.55 (0.52, 4.57) | 0.4288 |
| HH | 1.58 (0.78, 3.20) | 0.2034 |  | 1.75 (0.82, 3.76) | 0.1510 |  | 2.12 (0.75, 5.96) | 0.1557 |
| LH | 3.58 (1.55, 8.30) | 0.0029 |  | 3.87 (1.56, 9.55) | 0.0034 |  | 5.60 (1.70, 18.45) | 0.0046 |
| *P* for trend | 0.0013 |  |  | 0.0010 |  |  | 0.0043 |  |

Non-adjusted model: we did not adjust other covariates. Model 1: we adjusted age and female. Model 2: we adjusted variables which were significantly associated with outcomes of interest (*p* < 0.10) or changed the estimates of oxygen saturation on outcomes of interest by more than 10%.

HL, higher preoperative oxygen saturation and lower postoperative oxygen saturation; LL, lower preoperative oxygen saturation and lower postoperative oxygen saturation; HH, higher preoperative oxygen saturation and higher postoperative oxygen saturation; LH, lower preoperative oxygen saturation and higher postoperative oxygen saturation; mRS, modified Rankin Scale; OR, odds ratio.

**Figure I.** Flow chart of patient cohort


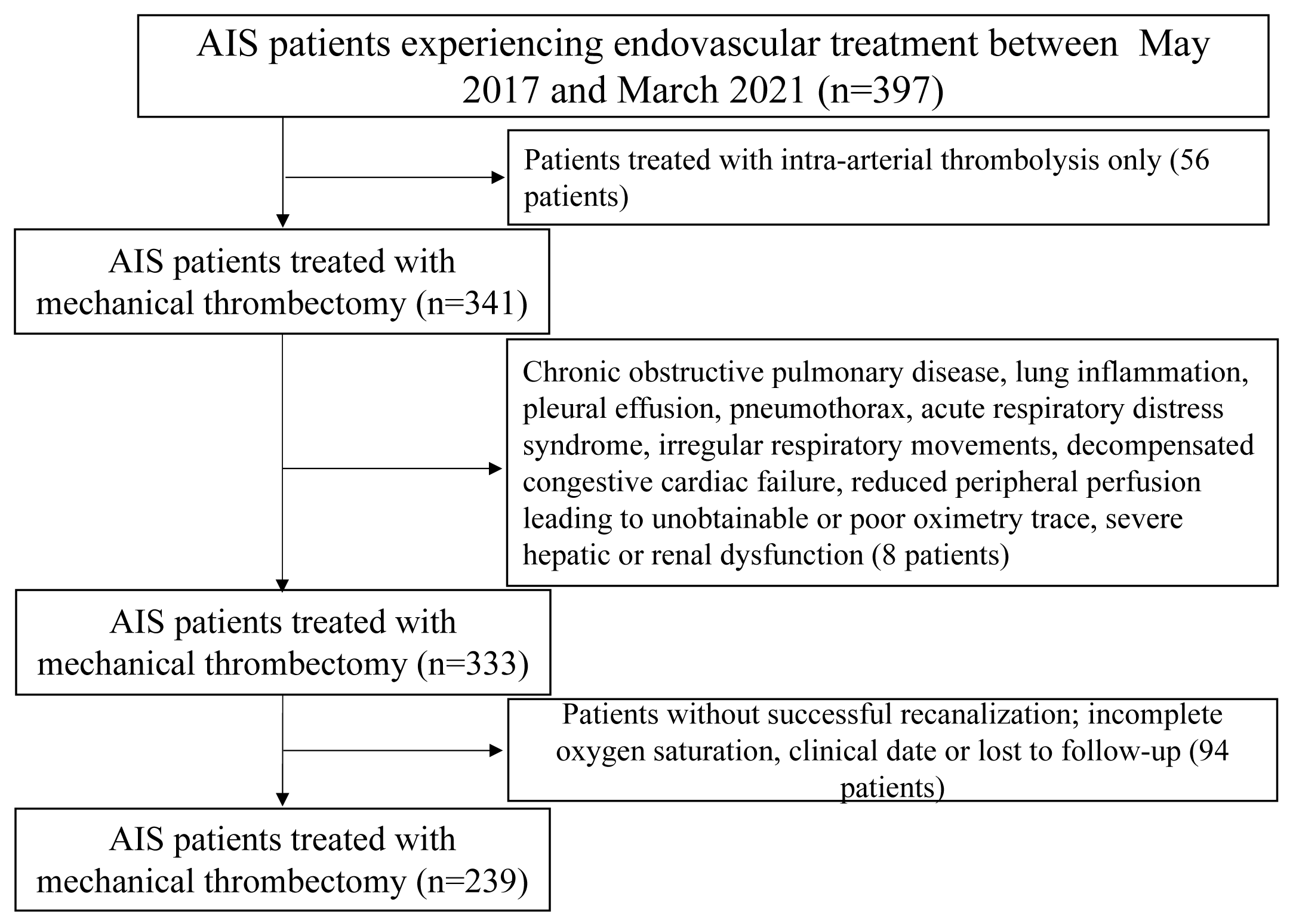


AIS: Acute ischemic stroke.
